# Response of phenological and agronomical attributes and thermal utilization efficiency of cotton cultivars to cumulative heat units under different sowing dates

**DOI:** 10.1186/s12870-025-08076-3

**Published:** 2026-01-21

**Authors:** Amany A. El-Ashmouny, Ahmed S.A. Hegab, Beelal A.A. Ali, Hani S. Saudy

**Affiliations:** 1 Cotton Physiology Department, Cotton Research Institute, Agricultural Research Center, Giza, Egypt; 2 Central Laboratory for Agricultural Climate (CLAC), Agricultural Research Center, Dokki, Egypt; 3 Agronomy Department, Faculty of Agriculture, Ain Shams University, Cairo, 11566 Egypt

**Keywords:** Cotton flowering, Fiber crops, Genotypic variations, Global worming, Thermal units, Light utilization

## Abstract

**Purpose:**

Rising temperatures resulting from climate change play a vital role in the development of crop phenological stages. Egyptian cotton (*Gossypium barbadense* L.) is an important industrial cash crop that requires optimal temperature for economic production. Extreme temperatures cause a sharp decline in cotton yield and quality via affecting plant growth and physiology. Sowing time is a fundamental practice for maximizing the crop’s genetic potential by minimizing the deleterious impacts of abiotic stress, specifically during critical reproductive stages, thereby enhancing the key yield attributes that collectively construct the final economic yield.

**Method:**

To create discrepancies in environmental growth resources over crop life span, three cotton cultivars (super Giza 94, Extra Giza 96 and Super Giza 97) were sown at three sowing dates (25-April, 15-May and 25-May) during 2022 and 2023 seasons. The meteorological data were collected and accumulated growing degree days (AGDD) were computed. Growth and phenological traits and yield attributes were estimated.

**Results:**

Findings exhibited that the lowest values of small boll shedding % and the highest values of earliness % were observed with Super Giza 94 when sown early on 25-April in both seasons. With sowing on 25-April, Super Giza 94 produced the maximum increases in all yield attributes. Super Giza 94 was the most efficient cultivar for exploiting the cumulative heat units under different sowing dates with a higher value at sowing on 25-April in both seasons.

**Conclusion:**

The proper choosing crop cultivar keeps one of the most serious decisions for cotton farmers, since it determines the ceiling of the potential return, upon which the other agricultural activities, such as sowing dates, can be tailored. Accordingly, the current work deduce that sowing Super Giza 94 cultivar on 25-april is regarded as a promising practice for efficient use of prevailing climatic factors, hence balanced vegetative growth with high cotton productivity.

## Introduction

Climate change has led to rising temperatures both locally and globally. As a result of climate changes, various environments in the world, especially arid ecosystems, are anticipated to suffer serious issues such as soil salinization, drought, and extreme temperatures [1–5]. These issues are regarded as critical abiotic stresses for agriculture development, threatening food security [6–10]. Under abiotic stresses, plant may suffer from nutrients deficiency due to a weak ability to uptake mineral, leading to a dysfunction an internal imbalance in nutrients [11–17]. Furthermore, environmental stress prompts the overproduction of hazardous molecules in plant cells, causing disruption to physiological and biological systems [18–22]. Therefore, crop growth, yield and quality are detrimentally affected under stressed-environments [23–28]. Specifically, climate change and the accompanying rise in temperature represent one of the greatest environmental, social, and economic threats facing the planet today. According to the IPCC [29], global surface temperature increased by approximately 1.1 °C above pre-industrial (1850–1900) levels in the last decade. From an agricultural perspective, projections indicate a temperature rise of 1.8–4 °C by the end of the 21st century, which could significantly disrupt cotton growth, development, and fiber production. Temperature influences every stage of cotton growth from seed germination to final crop production [30]. When temperatures exceed the optimal range (30/20°C), they accelerate evapotranspiration, trigger stomatal closure, and intensify water stress conditions, all of which negatively impact cotton crop [31, 32]. Studies indicated that cotton fiber yield declines by an average of 110 kg per hectare with each 1 °C increase in daily maximum temperatures. Extreme temperature events, known as short summer heat waves exceeding 5 °C above seasonal norms, severely impair plant growth and productivity by reducing daily photosynthesis and increasing nighttime respiration, leading to the consumption of stored capacities, resulting in increased square and boll shedding and reduced seed numbers/boll; such thermal stress compromises both fiber quality and overall crop yield [33, 34]. It has been stated that elevated nighttime temperatures adversely affect cotton plants by stimulating respiratory activity, which depletes energy reserves, reducing adenosine triphosphate (ATP) production in leaves, impairing carbohydrate storage, and ultimately diminishing cotton yield [35].

In the future, cotton growth is likely to be adversely influenced by the changing in climatic features encompassing temperature, light and humidity, therefore biomass accumulation and productivity may fluctuate considerably with the variability of climatic factors [36]. Frankly, it is difficult to accurately forecast the effects of climate change on crop productivity. However, it has be reported that the proper sowing date is a dynamic tool of climate change adaptation and has been exceedingly employed for efficiently utilization of heat and light by crop plants [37]. Delaying sowing may lead to temperature variations at various growth stages, influencing biomass production and productivity [38].

On the other site, developing crop cultivars is a significant tactic in climate change adaptability. Thus, cultivating cultivars adapted to climatic fluctuations is regarded as a crucial practice for sustaining crop productivity and quality under climate change-associated stresses. Due to the genetic variations among cotton cultivars, differences in their yield and quality under normal growth environments were assessed [39, 40]. However, cotton cultivars performances under variability of ecological factors need deep investigations.

The present work hypothesized that the potential tolerance of different cotton cultivars to environmental factors variability as sowing dates change may rely on the accumulated growing degree days (AGDD). Therefore, this study aimed to assess the relationship between phenological attributes and yield of three Egyptian cotton cultivars and three sowing dates based on AGDD, which could contribute to setting up an effective scenario for sustaining crop productivity under climate change.

## Materials and methods

### Trial area attributes

A 2-season field experiment was conducted in 2022 and 2023 at Sakha Research Station, Cotton Research Institute, Agricultural Research Center, Kafr El-Shiekh (31°18′30″N & 30°48′14″E), North Delta, Egypt. The study aimed to assess the dominance cumulative heat units under various sowing dates and their effects on phenology and yield attributes of cotton cultivars. The basal analysis illustrated that the experimental soil was clayey in texture, comprising sand (22.0%), silt (27.4%), clay (50.6%) with pH of 7.94, electrical conductivity of 1.90 dS m^–1^, organic carbon (1.55%), available nitrogen (15.1 mg L^− 1^), available phosphorus (11.2 mg L^− 1^), and available potassium (469 mg L^− 1^). The average monthly agro-climatic data of the study location during the two studied seasons (from April to November) are listed in Table 1. These data collected from automated weather station of Central Laboratory for Agricultural Climate (CLAC). The proceeding crop was clover in the first and second season.

**Table 1:** Average monthly agro-meteorological data of Sakha location during the seasons of 2022 and 2023

Weather factor	Air temperature (°C)		Relative humidity (%)	Soil temperature (°C), at depth of 20 cm	Wind Speed (m sec^− 1^)	Solar radiation (MJ m^− 2^ day^− 1^)
	Maximum	Minimum				
Season of 2022						
April	30.37	13.17	51.20	25.55	2.90	22.94
May	33.92	16.82	51.11	23.70	3.15	25.37
June	37.48	20.89	49.96	26.55	3.11	28.19
July	38.99	22.06	50.27	28.95	2.88	28.25
August	38.58	23.10	54.01	30.23	2.75	25.20
September	37.05	22.15	52.84	29.32	2.71	21.55
October	31.52	19.27	59.90	25.25	2.53	16.45
Season of 2023						
April	30.80	13.80	56.22	26.22	2.90	23.01
May	24.17	17.30	51.02	25.65	3.43	25.26
June	37.83	21.11	50.85	28.98	3.23	26.17
July	41.08	23.16	51.05	29.94	2.98	28.17
August	39.18	23.48	54.11	31.11	2.74	25.51
September	38.74	23.25	54.47	29.69	2.52	21.27
October	33.29	20.46	60.90	26.42	2.46	15.83

### Crop agro-management

Soil was enriched by single super phosphate (15.5% P_2_0_5_) at a rate of 55 kg P_2_0_5_ ha^− 1^ before sowing. Land was tailored via ridging and divided into plots having 5 rows with net size of 14.0 m^2^ (4.0 m length × 0.7 m width). Cotton seeds were manually drilled in rows on one side of the ridge with distance of 25 cm between hills. At 28 days after sowing (DAS), cotton seedlings were thinned to secure two plants per hill. Potassium fertilizer in the form of potassium sulfate (48.0% K_2_O) at a rate of 120 kg ha^− 1^ was applied 62 DAS. Nitrogen fertilizer in the form of ammonium nitrate (33.5% N) at a rate of 165 kg N ha^− 1^ was added in two equal portions 28 and 43 DAS.

### Trial design and treatments

The experiment employed a split plots design with four replicates, where the main plots involved three sowing dates and sup-plots were occupied by three cotton cultivars. To create different growth conditions a wide range of sowing dates was selected i.e., sowing on 25-April, 10-May, and 25-May in 2022 and 2023 seasons. The three tested cotton cultivars were Super Giza 94, Extra Giza 96 and Super Giza 97; and their fiber type, pedigree and origin are described in Table 2. The seeds of the tested cultivars were obtained from ton Research Institute, Agricultural Research Center, Giza, Egypt.

**Table 2:** Fiber type, pedigree and origin of tested cotton cultivars

Genotype	Fiber type	Pedigree	Origin
Super Giza 94	Super	10,229×Giza 86	Egypt
Extra Giza 96	Extra	G84×G70×G518×S62	Egypt
Super Giza 97	Super	(G89×A101)(G86)	Egypt

## Metrics

### Phenology and agronomic traits

The number of days from sowing until the first flower opens, the number of days from sowing until 50% of the flowers opens, the number of days from sowing until the first boll opens as well as the number of days from sowing until the second picking were counted.

At opening 50% of flowers, samples of six plants were randomly marked to record plant height and fruiting branches number plant^− 1^ with estimating leaf area plant^− 1^ [41]. Also, small boll shedding % (number of total bolls – number of open bolls / number of total bolls × 100), and earliness % were assessed.

At harvest (beginning of October), bolls number plant^− 1^, boll weight, seed index (weight of 100 seeds), seed cotton yield and lint % were measured.

### Accumulative growing degree days (AGDD)

Based on the obtained daily air temperatures from crop emergence till maturity (Table 1), growing degree days (GDD); called also cumulative heat units (CHUs) was estimated using formula 1 [42].

1 $$\:\mathrm{D}\mathrm{a}\mathrm{i}\mathrm{l}\mathrm{y}\:\mathrm{G}\mathrm{D}\mathrm{D}=\left[\left\{\mathrm{T}\mathrm{m}\mathrm{a}\mathrm{x}\:+\:\mathrm{T}\mathrm{m}\mathrm{i}\mathrm{n}\right)-\:\mathrm{T}\mathrm{b}\mathrm{a}\mathrm{s}\mathrm{e}/2\right]$$

Where:

Tmax and Tmin: daily maximum and minimum air temperatures, respectively,

Tbase: base temperature for cotton development which equals 12 °C [43]. This is the temperature below which no significant growth and development occurs [44].

Furthermore, thermal utilization efficiency based on total cumulative heat units (CHUs) and obtainable economic yield of cotton sown under different dates was computed by formula 2 [45].

2 $$\:\mathrm{T}\mathrm{h}\mathrm{e}\mathrm{r}\mathrm{m}\mathrm{a}\mathrm{l}\:\mathrm{u}\mathrm{t}\mathrm{i}\mathrm{l}\mathrm{i}\mathrm{z}\mathrm{a}\mathrm{t}\mathrm{i}\mathrm{o}\mathrm{n}\:\mathrm{e}\mathrm{f}\mathrm{f}\mathrm{e}\mathrm{c}\mathrm{i}\mathrm{e}\mathrm{n}\mathrm{c}\mathrm{y}=\:\frac{\mathrm{E}\mathrm{c}\mathrm{o}\mathrm{n}\mathrm{o}\mathrm{m}\mathrm{i}\mathrm{c}\:\mathrm{y}\mathrm{i}\mathrm{e}\mathrm{l}\mathrm{d}}{\mathrm{C}\mathrm{u}\mathrm{m}\mathrm{u}\mathrm{l}\mathrm{a}\mathrm{t}\mathrm{i}\mathrm{v}\mathrm{e}\:\mathrm{h}\mathrm{e}\mathrm{a}\mathrm{t}\:\mathrm{u}\mathrm{n}\mathrm{i}\mathrm{t}\mathrm{s}\:}\:\left(\mathrm{k}\mathrm{g}\:{}^{\circ}{\mathrm{C}}^{-1}\right)$$

### Statistical analysis

Data were statistically analyzed according to Gomez and Gomez [46], using SAS program software. To distinguish and compare the treatment means, the test of the least significant difference (LSD) at 0.05 probability level was employed.

## Results

### Vegetative traits

Cotton plant height, fruiting branches number plant^− 1^ and leaf area plant^− 1^ were significantly affected by sowing date and cultivar in both growing seasons of 2022 and 2023 (Table 3). The tallest cotton plants were produced from plots sown on 25-May, surpassing those sown on 25-April and 10-May in both seasons. On the contrary, sowing date on 25-April possessed the maximum fruiting branches and leaf area plant^− 1^, outperforming the sowing dates on 10-May and 25-May. Super Giza 94 was the potent cultivar for producing the highest values of plant height, fruiting branches number plant^− 1^ and leaf area plant^− 1^, outperforming Extra Giza 96 and Super Giza 97 in both seasons. As for interaction, sowing Super Giza 94 on 25-May resulted in the highest increase in plant highest in both seasons. While, Extra Giza 96 plots sown early on 25-April gave the shortest cotton plants. On the other hand, sowing on 25-April×Super Giza 94 was the effective combination for achieving the maximal values of fruiting branches and leaf area plant^− 1^ in both seasons.

**Table 3:** Influence of sowing date on plant height, fruiting branches number plant^− 1^ and leaf area plant^− 1^ of cotton cultivars estimating at opening 50% flowers in 2022 and 2023 seasons

Variable			Plant height (cm)		Fruiting branches number plant^− 1^		Leaf area plant^− 1^ (cm^2^)	
			2022	2023	2022	2023	2022	2023
Sowing date, S								
25-April			111.57	112.75	15.52	15.85	864.76	910.50
10-May			116.51	117.35	12.76	12.94	838.26	874.21
25-May			123.27	124.31	11.50	10.85	816.90	805.61
LSD_0.05_			0.15	0.22	0.27	0.12	2.78	2.57
Cultivar, C								
Super Giza 94			118.99	120.74	14.03	14.07	847.89	879.38
Extra Giza 96			115.19	115.71	12.55	12.26	834.93	848.09
Super Giza 97			117.17	117.96	13.19	13.31	837.09	862.85
LSD_0.05_			0.12	0.26	0.28	0.10	2.31	3.26
S×C								
25-April	Super Giza 94		113.46	114.58	16.83	16.95	877.27	920.40
	Extra Giza 96		109.86	111.07	14.40	14.67	853.00	902.17
	Super Giza 97		111.38	112.60	15.33	15.94	864.00	908.93
10-May	Super Giza 94		117.97	118.65	13.27	13.78	843.20	885.28
	Extra Giza 96		114.73	115.77	12.25	12.10	841.00	862.03
	Super Giza 97		116.82	117.63	12.75	12.95	830.57	875.31
25-May	Super Giza 94		125.54	128.98	12.00	11.49	823.20	832.47
	Extra Giza 96		120.97	120.29	11.00	10.01	810.80	780.07
	Super Giza 97		123.30	123.65	11.50	11.05	816.70	804.30
LSD_0.05_			0.20	0.45	0.48	0.18	4.00	5.64

### Shedding and earliness

**Fig. 1:**
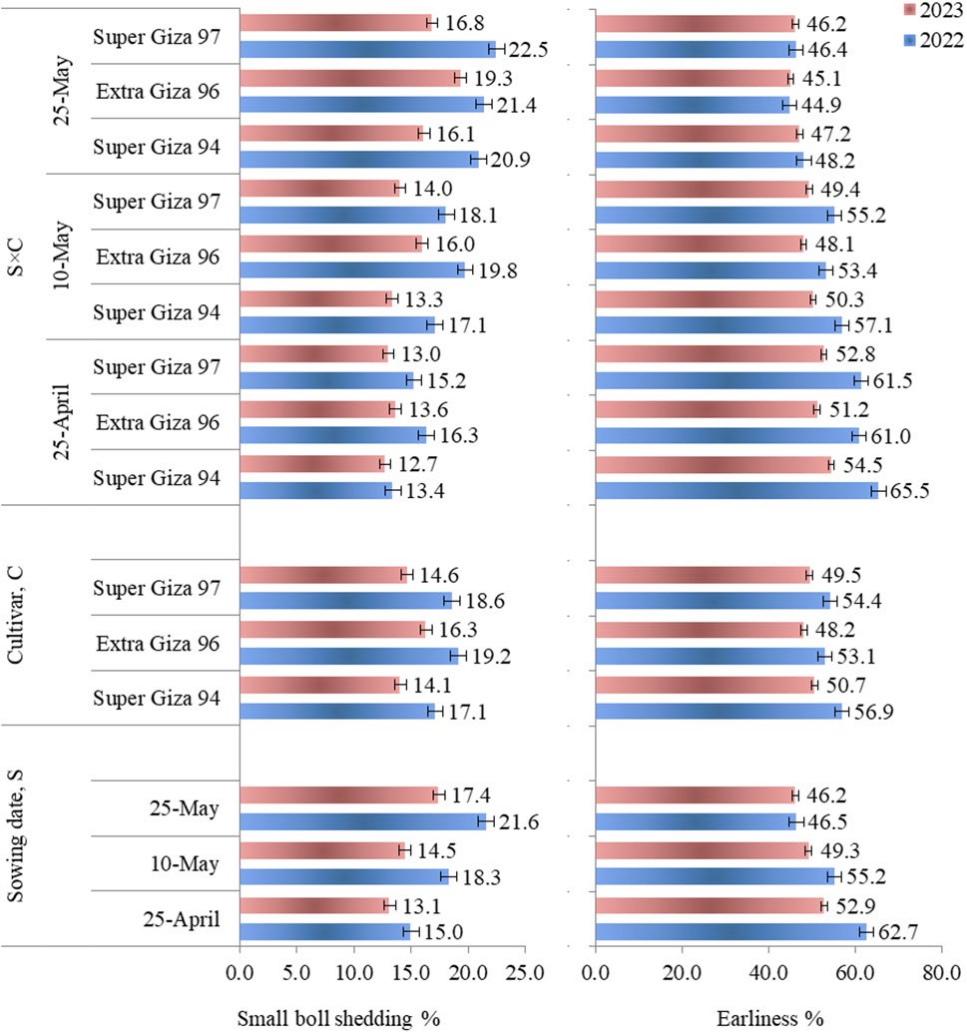
Small boll shedding % and earliness % of cotton as influenced by sowing date (S) and cultivars (C) and their interaction (C×S). LSD values for small boll shedding %: S = 0.31 and 0.33, C = 0.29 and 0.33, and C×S = 0.50 and 0.57 and 0.51 as well as for earliness %: S = 0.55 and 0.19, C = 0.50 and 0.17, and C×S = 0.86 and 0.29 in 2022 and 2023 seasons, respectively

Small boll shedding % increased and earliness % decreased with the delay in sowing time in 2022 and 2023 seasons (Fig. 1). Therefore, sowing on 25-May showed the maximum values of small boll shedding %, while the highest values of earliness % was obtained with sowing on 25-April. Super Giza 94 exhibited a lower small boll shedding % with a higher earliness % as compared to Extra Giza 96 or Super Giza 97 in both seasons. The lowest values of small boll shedding % and the highest values of earliness % were observed with Super Giza 94 when sown early on 25-April in both seasons.

### Yield attributes and lint %

As shown in Table 4, sowing dates had significant impacts on yield attributes of cotton in 2022 and 2023 seasons. Sowing on 25-April resulted in the maximum increases in open boll number plant^− 1^, boll weight, seed index and seed yield, surpassing the sowing dates on 10-May and 25-May. Also, sowing on 10-May outperformed sowing on 25-May in all studied yield attributes. Super Giza 94 was the superior cultivar for producing the highest values of open boll number plant^− 1^, boll weight, seed index and seed yield in both seasons. With sowing on 25-April Super Giza 94 produced the maximum increases in all yield attributes. Delaying sowing resulted in significant reductions in open boll number plant^− 1^, boll weight, seed index and seed yield in both seasons. Focusing on seed yield, it can be observed that delaying sowing date to 10-May and 25-May instead of 25-April, caused yield reductions amounted to 28.1 and 50.1% for Super Giza 94, 35.5 and 51.3% for Extra Giza 96 and 30.8 and 47.1% for Super Giza 97, respectively, as an average of the two seasons.

**Table 4:** Influence of sowing date on open boll number plant^− 1^, boll weight, seed index and seed yield of cotton cultivars in 2022 and 2023 seasons

Variable			Open boll number plant^− 1^		Boll weight (g)		Seed index (g)		Seed cotton yield (kg ha^− 1^)	
			2022	2023	2022	2023	2022	2023	2022	2023
Sowing date, S										
25-April			25.71	31.73	3.20	3.18	12.49	12.47	3242.4	3581.0
10-May			21.57	25.24	2.61	2.58	11.94	12.06	2132.8	2565.2
25-May			15.20	18.65	2.05	2.28	11.20	11.20	1361.9	2114.1
LSD_0.05_			0.56	0.54	0.04	0.06	0.24	0.15	48.7	59.9
Cultivar, C										
Super Giza 94			21.78	27.67	2.82	2.88	12.18	12.26	2497.7	2927.5
Extra Giza 96			19.91	22.88	2.43	2.52	11.61	11.57	1927.9	2588.9
Super Giza 97			20.79	25.07	2.61	2.63	11.85	11.90	2311.5	2743.8
LSD_0.05_			0.43	0.52	0.03	0.04	0.12	0.12	52.4	37.5
S×C										
25-April	Super Giza 94		26.33	34.86	3.42	3.43	12.90	12.93	3621.0	3711.0
	Extra Giza 96		24.83	29.00	3.00	3.00	12.00	11.90	2863.8	3459.8
	Super Giza 97		25.97	31.33	3.17	3.10	12.57	12.57	3242.4	3572.3
10-May	Super Giza 94		23.08	27.33	2.77	2.73	12.13	12.35	2489.0	2785.1
	Extra Giza 96		20.50	23.44	2.44	2.47	11.83	11.80	1780.5	2312.8
	Super Giza 97		21.13	24.95	2.62	2.53	11.87	12.02	2129.1	2597.7
25-May	Super Giza 94		15.94	20.83	2.27	2.47	11.50	11.50	1383.1	2286.5
	Extra Giza 96		14.40	16.19	1.85	2.10	11.00	11.00	1139.5	1994.2
	Super Giza 97		15.27	18.92	2.03	2.27	11.10	11.10	1563.1	2061.6
LSD_0.05_			0.74	0.91	0.05	0.07	0.21	0.21	89.9	63.7

**Fig. 2:**
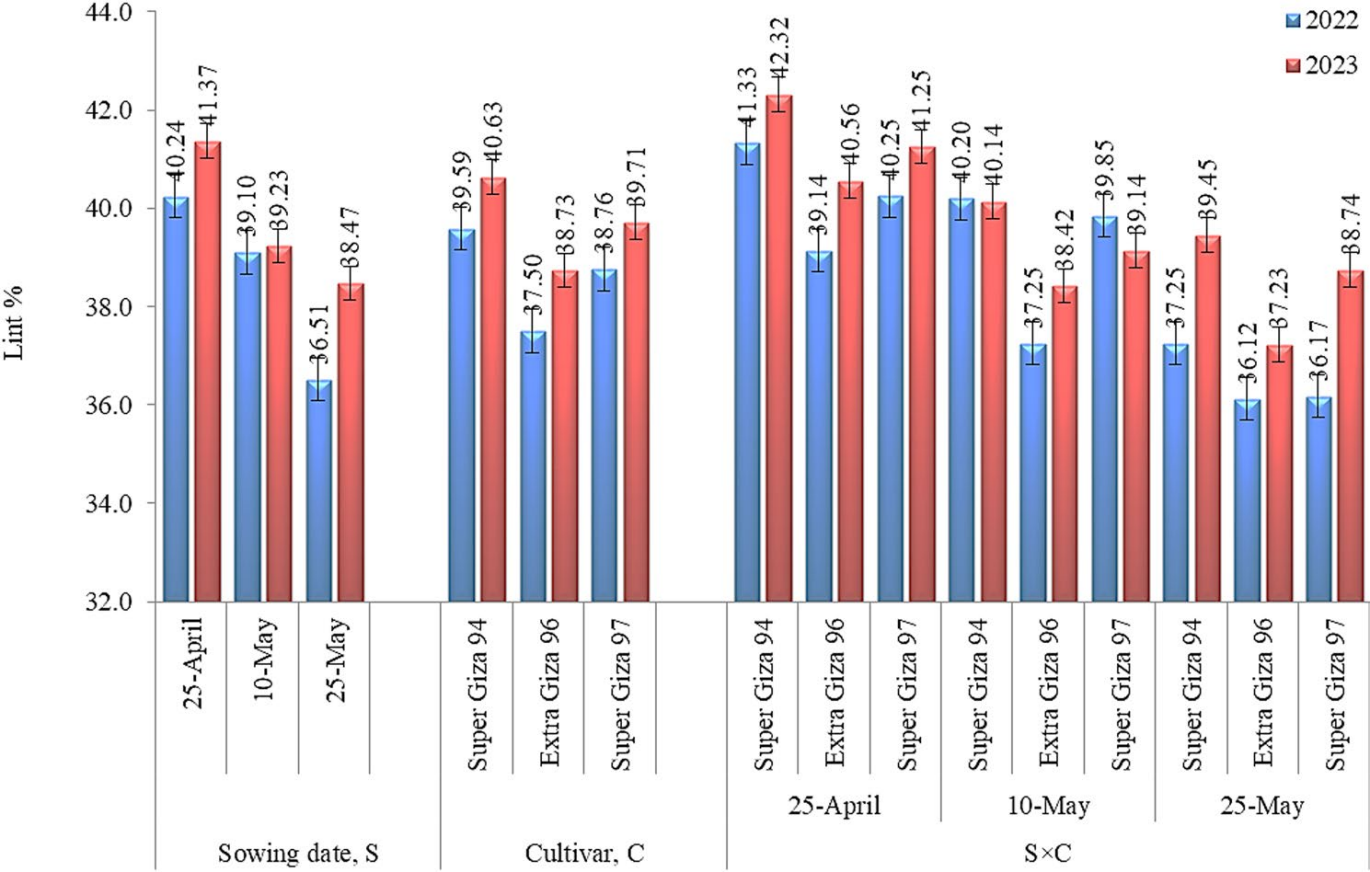
Lint % of cotton as influenced by sowing date (S) and cultivars (C) and their interaction (C×S). LSD values: S = 0.56 and 0.45, C = 0.73 and 0.33, and C×S = 0.23 and 0.51 in 2022 and 2023 seasons, respectively

Concerning lint %, Fig. 2 illustrated that sowing date and cultivar and their interaction markedly influenced cotton lint % in 2022 and 2023 seasons. In this regard, delaying sowing date led to decreasing lint %, where the lowest values were obtained with 25-May. Sowing on 25-April showed greater increases of 1.02 and 1.10 times in 2022 season and 1.05 and 1.07 times in 2023 season compared to sowing on 10-May and 25-May, respectively. Super Giza 94 recorded the highest lint %, surpassing Extra Giza 96 by 1.05 and 1.02 times and Super Giza 97 by 1.04 and 1.02 times in the first and second seasons, orderly. The maximum values of lint % were achieved with the combination of sowing on 25-April×Super Giza 94 outperforming the other possible interactions of sowing dates and cultivars in both seasons. All cultivars showed remarkable reduction in lint % as sowing date delayed than normal.

### Relation between AGDD, phenology and yield of cotton

The variations in cotton cultivars phenological stages expressed in number of day from sowing (NDFS) until specific growth stage and AGDD due to changing sowing dates in 2022 and 2023 seasons are presented in Table 5. Obviously, data generally revealed that delaying sowing leads to reducing the growth durations of later phenological stages (number of days from sowing until the first boll opens and number of days from sowing until the second picking). The total AGDD values for each cultivar were higher with sowing on 25-April than that of sowing on 10-May and 25-May. Under each sowing date, Super Giza 97 plants accumulated greater total AGDD than that of Super Giza 94 and Extra Giza 96.

**Table 5:** Number of days from sowing (NDFS) and accumulated growing degree days (AGDD) for phenological stages of cotton cultivars under different sowing dates in 2022 and 2023 seasons

Variable			NDFS until the first flower opens	NDFS until 50% of the flowers opens	NDFS until the first boll opens	NDFS until the second picking	Total AGDD
Season of 2022							
25-April	Super Giza 94	NDFS	58	80	123	150	
		AGDD	650	968	1631	1852	5101
	Extra Giza 96	NDFS	65	80	130	160	
		AGDD	708	926	1691	1911	5236
	Super Giza 97	NDFS	62	77	127	156	
		AGDD	968	750	1736	1948	5402
10-May	Super Giza 94	NDFS	60	81	90	152	
		AGDD	774	1091	1227	1474	4566
	Extra Giza 96	NDFS	66	81	95	162	
		AGDD	858	1091	1304	1538	4791
	Super Giza 97	NDFS	66	82	97	157	
		AGDD	858	1106	1334	1599	4897
25-May	Super Giza 94	NDFS	55	72	85	143	
		AGDD	773	1039	1234	1470	4516
	Extra Giza 96	NDFS	58	72	87	150	
		AGDD	772	1010	1234	1485	4501
	Super Giza 97	NDFS	55	70	85	148	
		AGDD	822	1039	1264	1543	4668
Season of 2022							
25-April	Super Giza 94	NDFS	58	85	118	168	
		AGDD	769	1280	1728	2395	6172
	Extra Giza 96	NDFS	66	93	123	173	
		AGDD	831	1311	1787	2396	6325
	Super Giza 97	NDFS	61	89	120	170	
		AGDD	845	1341	1784	2471	6441
10-May	Super Giza 94	NDFS	60	90	119	169	
		AGDD	782	1249	1758	2375	6164
	Extra Giza 96	NDFS	65	94	122	175	
		AGDD	784	1295	1795	2421	6295
	Super Giza 97	NDFS	61	91	121	171	
		AGDD	868	1326	1801	2405	6400
25-May	Super Giza 94	NDFS	55	83	115	165	
		AGDD	632	1044	1588	2335	5599
	Extra Giza 96	NDFS	60	88	118	168	
		AGDD	675	1112	1619	2357	5763
	Super Giza 97	NDFS	58	87	117	167	
		AGDD	745	1187	1666	2390	5988

**Fig. 3:**
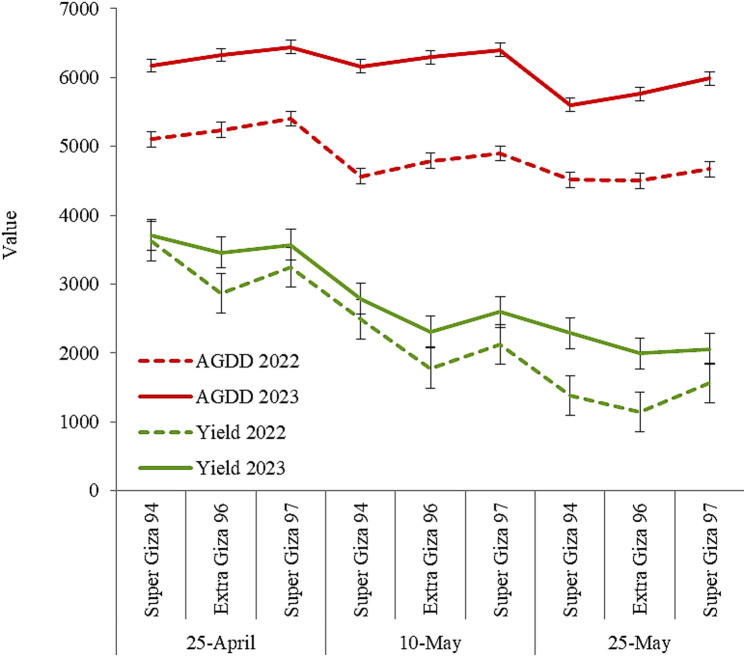
Relationship between yield and accumulated growing degree days (AGDD) of cotton cultivars under different sowing dates in 2022 and 2023 seasons

**Fig. 4:**
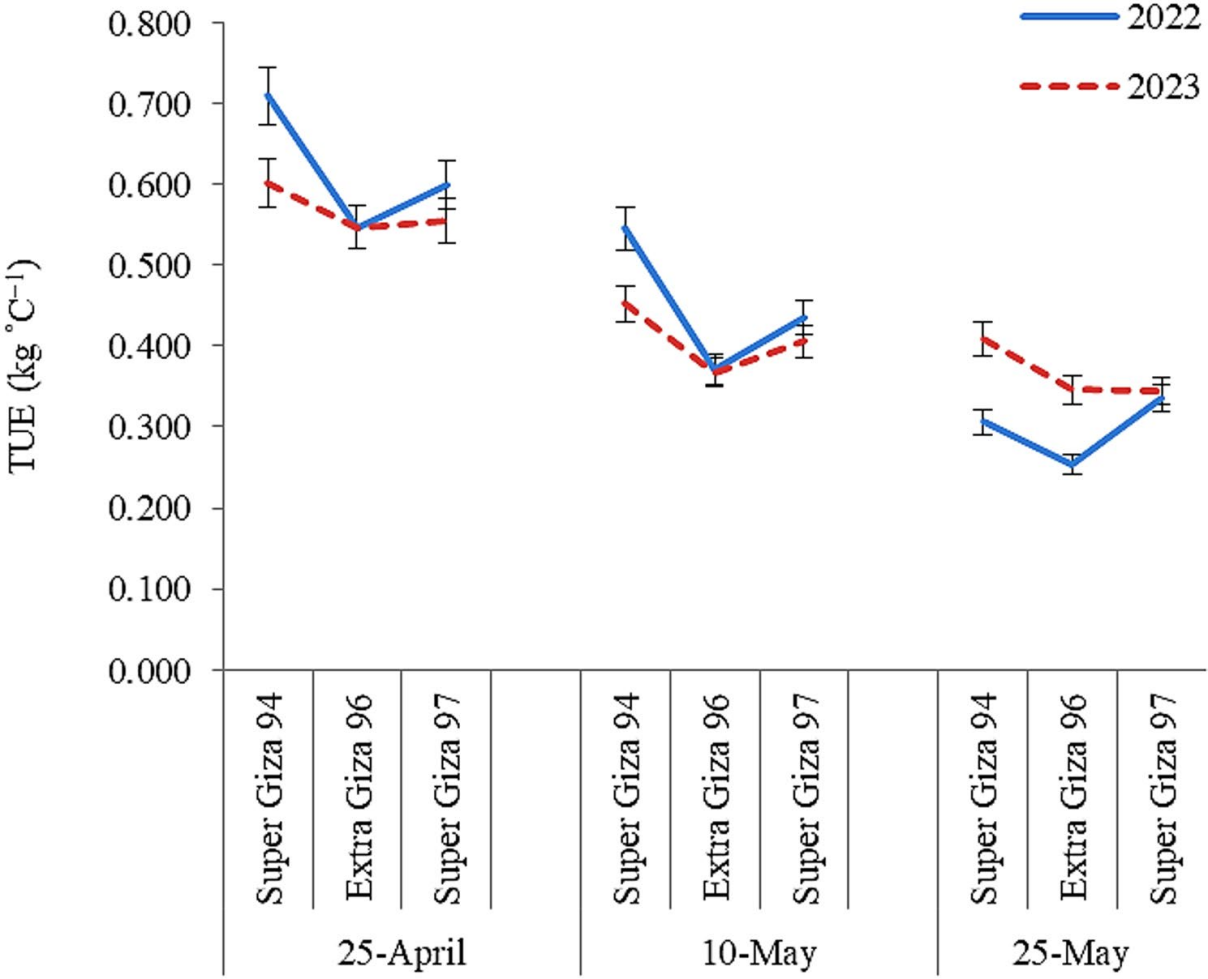
Thermal utilization efficiency (TUE) of cotton cultivars under different sowing dates in 2022 and 2023 seasons

Relationships between AGDD from sowing till harvesting date and yield of cotton cultivars under different sowing dates are presented in Fig. 3. Despite Super Giza 94 plants accumulated lower AGDD, they produced the highest seed cotton yield compared to the other two cultivars along different sowing dates in both seasons. While, Super Giza 97 plants accumulated higher AGDD with lower seed cotton yield compared to Super Giza 94 along different sowing dates in both seasons. By computing the thermal utilization efficiency (Fig. 4), the outputs revealed that Super Giza 94 was the most efficient cultivar for exploiting the CHUs under different sowing dates with higher values when sown on 25-April in both seasons.

## Discussion

Reports indicate that heat stress, as a factor of climate change, causes significant disturbances in plant physiology and biochemistry, manifested in a decrease in photosynthesis, photosynthetic pigments, and dry matter accumulation, which negatively affects the morphological features of Egyptian cotton genotypes [47]. It has been reported that many metabolic processes related to plant growth have been disrupted due to exposure to stresses [48–52]. This may be attributed to the fact that abiotic stress stimulates the excessive formation of reactive oxygen species, which are molecules that impair plant cell functions [53–55]. Herein, pigment formation and photosynthesis apparatus [56–58], stomatal performance [59, 60], nutrients homeostasis [61, 62], and antioxidant activity [63, 64] were greatly affected as subjecting to abiotic stresses. The development of crop storage organs (sink) can be influenced either by assimilates gained by leaves as a photosynthetic structure (source) or by the potential for photosynthesis utilization [65]. The balance between source and sink could be disturbed by subjecting to unfavorable conditions or stresses. Avoiding the unfavorable climatic conditions such as unsuitable temperature could be achieved by sowing in appropriate times. Dominance of unfavorable conditions during crop growth may adversely influence growth and development [66]. The current study revealed that early sowing date (25-April) performed suitable climate conditions thus improves vegetative growth characters, lint % and earliness % with enhancing open boll number plant^− 1^, boll weight, seed index and cotton seed yield. While, declines in growth and yield attributes were more evident with delaying sowing up to 10th May. Delaying sowing date forced plants to reduce vegetative growth period, hence decreased yield was obtained [67]. Owing to the remarkable late sowing-associated declines in leaf are index and seed index, seed yield reduced [68]. The enhanced vegetative growth under suitable early-season climate conditions is consistent with the physiological mechanisms [69].

Changes in temperature and solar radiation as climatic factors have remarkable influences on crop growth [70]. The efficient use of growth resources is a precondition for improving crop yields [71]. The dominance of high temperatures at growth starting and at maturity may have a beneficial effect on yield [72]. The future trend of rising annual temperatures and elevating frequency of high temperatures in a warm climates may hasten cotton growth and development, accelerating maturity and partaking in increased yield [73, 74]. However, elevating the frequency of high-temperature days along growing season may adversely affect cotton growth and development [72]. In this respect, thanks to sowing on 25-April, the growth stages of the cotton plant coincided with the appropriate rise in temperatures, especially during the reproduction stage, which stimulated the rate of boll opening and the efficient use of growth resources, hence improved the cotton yield [75]. Contrariwise, cotton crop sown late was significantly lower than that sown early, probably because late sowing resulted in exposure to hotter temperatures as well as longer-lasting diseases and pests [76]. Moreover, prolonged periods of high temperatures can depress the activity of photosynthesis, increase the rates of transpiration, reduce nutrient utilization, and make cotton more sensitive to water deficiency, influencing boll formation and setting, leading to losses in yield [77]. Additionally, in late-sowing, cotton plants speedily enter the boll formation stage; thus prolonged and excessively high temperatures have a detrimental impact on activity of pollens and fruit development [78]. Maturity of fruits is closely linked to temperature changes, which play a key role in crop growth rate and ripening time [79]. The effect of sowing dates is not only related to temperature but also to changes in lighting conditions. Proper light and temperature circumstances had a distinctive potentiality to improve bolls number and weight of cotton per unit area, thereby enhancing the economic yield [80]. Accordingly, the current study exhibited that yield attributes of early-sown cotton were higher than those of mid- or late-sown cotton. This may be ascribed to the fact that early-sown cotton plants enter the growth stage earlier under adequate light conditions, which helps in the formation and development of cotton bolls earlier [80]. On the contrary, sowing of cotton on 10-May (mid-sown) and 25-May (late-sowings) may face a shorter growing period, weakening the ability to achieve the progress in growth and yields of 25-April (early sown) [81]. This result is consistent with the work of El-Lattief [82], who reported a direct positive correlation between early sowing dates and increased number of harvested bolls in Egyptian cotton. An extended fruit development period under favorable conditions is paramount for achieving superior boll size and weight [83]. Late planting exposes cotton to heat stress during reproductive development, severely limiting yield potential by negatively impacting boll retention and fiber quality [84].

Concerning the differences among cotton cultivars, Super Giza 94 exhibited superiority in growth traits and yield attributes as compared to Extra Giza 96 and Super Giza 97. Plants of Super Giza 94 were taller, while Extra Giza 96 had more compacted growth habit [85, 86]. Super Giza 94 exhibited the most vigorous vegetative growth and highest earliness index [87, 88]. This variation in plant architecture is a key distinguishing morphological trait among modern Egyptian cotton varieties [89]. Owing to genetic predisposition of Super Giza 94, better growth and yield components were obtained [90, 91]. The correlation between enhanced leaf area as growth trait and reduced boll shed as reproductive trait of superior genotype can be explained by the greater photosynthetic capacity for supporting fruits formation [92]. Thus, the significant yield advantage of Super Giza 94 is a cumulative result of its genetic ability to produce more bolls, each with a greater mass, containing heavier seeds than other cultivars. The genetic predisposition for higher boll number and weight in Super Giza 94 was previously highlighted [90]. The positive correlation between boll number, boll weight, and final seed cotton yield is a well-established physiological principle [93], which mainly ascribed to variations in genotypes potentiality. In addition to the differences in growth habits, the variability of cultivar to utilize the growth resources such as soil nutrients and climatic factors has a critical act for determining the productivity and quality [94–96]. Under limited growth resources, different genotypes showed different genetic potentiality in utilization of nutrients and thermal units [97–99]. Physiologically, the ability of cultivars to withstand stress depends primarily on their ability to maintain high photosynthetic efficiency, modulate osmotic potential, and raise the activity of various antioxidant defense patterns [99–101].

The estimation of relevance between accumulated growing degree days (AGDD) and final seed cotton yield reveals critical insights into the genetic efficiency of the tested cultivars. The results demonstrated that a higher AGDD does not automatically translate to a higher yield; instead, the efficiency with which a cultivar converts thermal units into harvestable product is a key determinant of productivity. The superiority in yield of Super Giza 94, achieved with the lowest AGDD, is a hallmark of a highly efficient and likely earlier-maturing cultivar. This suggests that Super Giza 94 progresses through its phenological stages (from sowing to harvest) more rapidly and/or is more effective at partitioning photo-assimilates into reproductive structures (bolls) during its growth cycle. Super Giza 94 can establish a canopy and set a significant boll load before the accumulation of excessive heat units, which can often be associated with late-season stress. This high thermal efficiency means farmers can potentially achieve top yields with a shorter field occupation time, reducing exposure to late-season risks. This aligns with the principles discussed by Loka and Oosterhuis [33], who note that genetic advances in cotton often focus on improving the conversion efficiency of environmental resources, like heat units, into seed cotton yield. Conversely, the combination of the highest AGDD and lowest grain yield in Extra Giza 96 indicates poor thermal utilization efficiency. This cultivar likely has a longer maturity period or suffers from higher rates of fruit abscission (shedding) under the accumulated heat. The extra thermal time did not contribute to additional yield but may have been expended on extended vegetative growth or lost through stress-induced shedding. This genotype’s requirement for more heat units to produce fewer yields makes it less suitable for production systems where maximizing efficiency is a goal. This negative relationship between excessive AGDD and yield in less adapted varieties has been observed in previous studies [102], which highlighted how developmental timing relative to temperature is crucial. Conclusively, these results powerfully illustrate that yield is a function of both genetics and environment, measured here as thermal time. Super Giza 94 demonstrates itself to be a superior cultivar not just because of its high yield, but because of its highly efficient use of the available growth resources. This characteristic is increasingly important in the context of climate change, where maximizing output per unit of environmental input is essential. The performance of Extra Giza 96 serves as a reminder that longer duration does not guarantee higher yield and that selecting cultivars for specific environments must consider their thermal efficiency [103].

## Conclusions

It is extremely important to assess the optimal cotton sowing date that is adaptable to future potential climate changes. The appropriate sowing date ensures that the different phenological stages coincide with prevailing weather conditions, especially temperature and light. In this respect, the findings of the present research exhibited that for regulating the phenological responses in favor of high productivity, cotton should be sown on 25th April. Also, in order to make more efficient utilization of environmental resources, it is recommended to cultivate Super Giza 94 due to its high ability to use temperatures and light compared to other tested varieties, which leads to a higher cotton yield. Thus, Super Giza 94 could be considered a benchmark cultivar for high cotton production systems in Egypt. Despite this investigation mainly focuses on cotton sowing in North Delta of Egypt, the obtained findings are commonly practicable, especially in areas with similar meteorological features. Given the uncertainty surrounding climate changes, there is an urgent need in the future researches to employ meteorological forecasting data and create climate models to determine the optimal sowing date for cotton to optimize the utilization efficiency of heat and light resource, and thus maintain productivity.

## Data Availability

The datasets used and/or analyzed during the current research are available from the corresponding author upon reasonable request.
